# In the Limelight: Photoreceptors in Cyanobacteria

**DOI:** 10.1128/mBio.00741-16

**Published:** 2016-06-28

**Authors:** Devaki Bhaya

**Affiliations:** Carnegie Institution for Science, Stanford, California, USA

## Abstract

Certain cyanobacteria look green if grown in red light and vice versa. This dramatic color change, called complementary chromatic adaptation (CCA), is caused by alterations of the major colored light-harvesting proteins. A major controller of CCA is the cyanobacteriochrome (CBCR) RcaE, a red-green reversible photoreceptor that triggers a complex signal transduction pathway. Now, a new study demonstrates that CCA is also modulated by DpxA, a CBCR that senses yellow and teal (greenish blue) light. DpxA acts to expand the range of wavelengths that can impact CCA, by fine-tuning the process. This dual control of CCA might positively impact the fitness of cells growing in the shade of competing algae or in a water column where light levels and spectral quality change gradually with depth. This discovery adds to the growing number of light-responsive phenomena controlled by multiple CBCRs. Furthermore, the diverse CBCRs which are exclusively found in cyanobacteria have significant biotechnological potential.

## COMMENTARY

Light is critical for any organism that relies on photosynthesis for growth. However, light intensity and quality can vary dramatically in both terrestrial and aquatic environments, which can significantly impact photosynthetic efficiency. Thus, it is no surprise that organisms have evolved a host of exquisitely tuned photoreceptors to sense and respond to the intensity, quality, and directionality of light. Members of the ancient phylum *Cyanobacteria*, which are considered the evolutionary precursor of algal and plant plastids, encode a vast array of such photosensors, including the phytochromes (1, 2).

Phytochromes occur in plants, algae, nonphotosynthetic bacteria, and fungi but have not been identified in archaea or in animals (3–5). They constitute a large superfamily of GAF domain-containing photoreceptors that use bilins as their chromophore. Typically, they absorb light in two regions of the visible spectrum, which stimulates the interchange between stable photoconvertible isomers. The canonical phytochrome, first identified in plants, absorbs red light in the Pr state, which converts it to the Pfr state. The Pfr state can revert to Pr by the absorption of far-red light or by a dark reversion step. These different photocycle states of phytochrome, in turn, control various signal transduction pathways.

The phytochrome superfamily has been divided into three subfamilies based on domain structure (4). Group I, best characterized in plants, contains the canonical phytochromes that have the unusual “knotted” PAS-GAF-PHY tridomain structure (3). Group II contains GAF-PHY domains but lacks the PAS domain (6). Cyanobacteria contain examples of both group I and II photosensors, but these are less well characterized. Group III is comprised of cyanobacteriochromes (CBCRs), which are exclusively found in cyanobacteria. CBCRs represent an abundant and diverse subfamily that contains bilin-binding PAS-GAF domains. Remarkably, the CBCRs can be activated by a diverse set of wavelengths that cover almost the entire spectrum from UV to near infrared (1, 7, 8). This unexpected and exciting discovery has opened up active fields to probe how molecular structure can influence absorption spectra (6) and in understanding the roles of these various CBCRs in controlling cellular processes.

**FIG 1:**
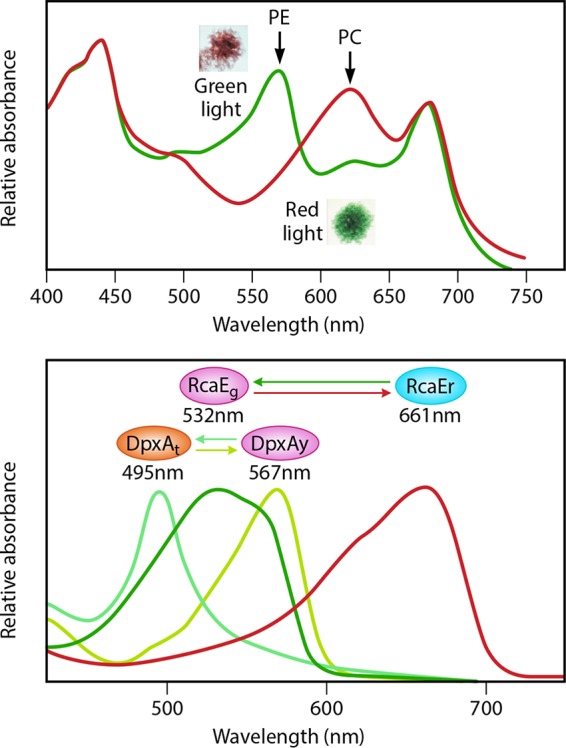
(Top) Whole-cell absorption spectra of *F. diplosiphon* cells. Cells grown in green light accumulate PE and look brick red (inset), while cells grown in red light look green (inset) and accumulate PC. The maximum absorption peaks of PE (λ_max_, 540 nm) and PC (λ_max_, 620 nm) are shown by arrows. (Bottom) Absorption spectra of DpxA_t_ (teal; λ_max_, 495 nm), DpxA_y_ (yellow; λ_max_, 567 nm), RcaE_g_ (green; λ_max_, 532 nm), and RcaE_r_ (red; λ_max_, 661 nm). Ovals represent the different forms of DpxA and RcaE with their peak absorption wavelengths shown below. The interconversion between these forms is shown by colored arrows. The composite figure is adapted and slightly modified (with permission) from figures that appear in references 9 and 14.

One of the most striking examples of light-regulated control by CBCRs is a phenomenon known as complementary chromatic adaptation (CCA), in which certain cyanobacteria can appear in different colors based on the light in which they are grown (Fig. 1). CCA was observed as early as 1902 by Engelmann and others and is best characterized in the filamentous cyanobacterium *Fremyella diplosiphon*, in which cells appear green if grown in red light and vice versa (9). This change in pigmentation represents a change in the composition of the multiprotein light-harvesting phycobilisome (PBS). PBS proteins are a major constituent in the cell that allows cells to optimize absorption of ambient light for photosynthesis under various light conditions. This, in turn, can provide cells a growth advantage.

It turns out that there are different types of CCAs and that the control of CCA is complex and regulated at many levels. Typically, cells that exhibit CCA can alter the composition of the PBS in response to light quality (9). In *F. diplosiphon*, the levels of PBS pigmented polypeptides phycoerythrin (PE; λ_max_, 540 nm) and phycocyanin (PC; λ_max_, 620 nm) were shown to change based on ambient light conditions. In a series of elegant experiments performed in the laboratory of Grossman and others, it was demonstrated that changes in PBS composition were controlled at the transcriptional level (10, 11).

This discovery spurred the hunt for the regulators of CCA. Elegant analyses of the *rca* (regulator for chromatic adaptation) pathway that exploited “color” mutants in *F. diplosiphon* uncovered regulatory components that included RcaE as well as two response regulators, RcaF and RcaC*.* RcaE was the first “phytochrome-like” photoreceptor discovered in bacteria (12, 13), although it is now classified as a CBCR (it has a GAF and a PAS domain). RcaE, like plant phytochromes, contains a cysteine in the GAF domain that covalently binds a chromophore (bilin or a linear tetrapyrrole), but unlike plant photoreceptors, which typically respond to red/far-red light, RcaE is sensitive to green and red light. The complex signal transduction cascade triggered in red or green light suggests that RcaE, which has a histidine kinase domain in addition to the GAF domain, can differentially phosphorylate RcaF and RcaC. These response regulators, in turn, activate several operons responsible for the production of PBS components. The result of this regulatory cascade is a PBS composition that has been altered in response to different light conditions (11).

Now, Wiltbank and Kehoe (14) have shown that another CBCR, called DpxA (decreased phycoerythrin expression), also plays a key role in the regulation of CCA in *F. diplosiphon*. Interestingly, DpxA has very different light-sensing properties than RcaE; it can respond to yellow (λ_max_, 568 nm) and teal (λ_max_, 494 nm) light. Using a *dpxA* mutant along with spectral analysis and measurements of kinase activity, DpxA was shown to repress the accumulation of PE, the major PBS component that makes cells look red. Thus, as expected, a null mutant of DpxA looks black when grown in white light because of elevated PE levels, in combination with normal levels of PC (in comparison, wild-type cells look green). The authors also demonstrated that DpxA_T_ (produced when cells are irradiated with yellow light) is the active form of the photoreceptor that represses PE accumulation while DpxA_Y_ is the inactive form of the photoreceptor.

In essence, this two-sensor system provides a sophisticated degree of control of CCA. By having two CBCRs, RcaE and DpxA, which are responsive to different wavelengths, the organism has evolved to sense a wide spectrum of light. This sensory information is transduced via a signaling pathway to tightly control PE expression across almost all light wavelengths. Under red light, RcaE is the major regulator controlling PBS composition, by activating PC expression and repressing PE expression. DpxA plays a minor role under these conditions. However, in yellow light, RcaE is in a less active state and DpxA plays the major role of repressing PE expression by driving DpxA_Y_ to the active form DpxA_T_. This leads to a model in which RcaE plays the role of a “coarse-control knob” under both red and green light that impacts both PE and PC levels. On the other hand, DpxA, which responds to a narrower range of wavelengths (between yellow and teal), affects only PE, thus acting like a “fine-control knob.”

The discovery of a two-system control of CCA brings up several interesting points. DpxA is the first yellow-light-sensing CBCR identified with a specific function in modulating CCA. However, based on sequence homology, there are several other DpxA-like proteins in cyanobacteria, whose roles are yet to be discovered. The presence of two (or more) light-sensing systems raises the question of the role of such systems in the environment. Initial characterizations of CBCRs are normally carried out using defined narrow wavelengths, but this method does not represent the natural environment. Over the course of the day, in water columns or in shaded situations, wavelengths and intensities can change dramatically. *F. diplosiphon* grows in aquatic environments; water attenuates red light, and so with depth, the green-to-red ratio increases, and there may also be other shading effects. The ability to finely modulate levels of PBS would provide a growth fitness advantage to the population (15). This phenomenon is reminiscent of “phenotypic plasticity” noted in eukaryotes and bacterial cells, whereby they exhibit two or more distinct morphological states in response to transient changes in the environment (16). Clearly, having such a wide range of CBCRs provides cyanobacteria enormous flexibility in tuning cellular responses to light (or possibly to other cues, such as redox conditions). Indeed, some cyanobacterial species have several such CBCRs and different chromophores (17–20). Potential cross talk and networks of control among CBCR pathways represent new frontiers in our understanding of “photoresponsivity” in photosynthetic organisms.

The focus of this commentary has been on the novel regulatory roles of multiple CBCRs in CCA (21), but these versatile photosensors have also been implicated in the regulation of phototaxis (22, 23), cell aggregation via cyclic di-GMP (24), and cyclic AMP (cAMP) levels (25). As more cyanobacterial species are examined, this palette of functions is likely to grow more diverse. Finally, the great versatility of these CBCRs and their obvious potential in building new optogenetic tools make them worthy of sustained attention at the level of basic and applied research (26, 27).
